# A Comprehensive Review of Pain Interference on Postural Control: From Experimental to Chronic Pain

**DOI:** 10.3390/medicina58060812

**Published:** 2022-06-16

**Authors:** Frédéric J. F. Viseux, Martin Simoneau, Maxime Billot

**Affiliations:** 1 Centre d’Evaluation et de Traitement de la Douleur (CETD), Hôpital Jean Bernard, Centre Hospitalier de Valenciennes, F-59322 Valenciennes, France; 2 Département Sciences de l’Homme et du Vivant (SHV), Université Polytechnique Hauts-de-France (UPHF), LAMIH, CNRS, UMR 8201, F-59313 Valenciennes, France; 3 Département de Kinésiologie, Faculté de Médecine, Université Laval, Québec, QC G1V 0A6, Canada; martin.simoneau@kin.ulaval.ca; 4 Centre Interdisciplinaire de Recherche en Réadaptation et Intégration Sociale (CIRRIS) du CIUSSS de la Capitale Nationale, Québec, QC G1M 2S8, Canada; 5 PRISMATICS Lab (Predictive Research in Spine/Neuromodulation Management and Thoracic Innovation/Cardiac Surgery), Poitiers University Hospital, F-86000 Poitiers, France; maxime.billot@chu-poitiers.fr

**Keywords:** pain, postural control, rehabilitation

## Abstract

Motor control, movement impairment, and postural control recovery targeted in rehabilitation could be affected by pain. The main objective of this comprehensive review is to provide a synthesis of the effect of experimental and chronic pain on postural control throughout the available literature. After presenting the neurophysiological pathways of pain, we demonstrated that pain, preferentially localized in the lower back or in the leg induced postural control alteration. Although proprioceptive and cortical excitability seem modified with pain, spinal modulation assessment might provide a new understanding of the pain phenomenon related to postural control. The literature highlights that the motor control of trunk muscles in patient presenting with lower back pain could be dichotomized in two populations, where the first over-activates the trunk muscles, and the second under-activates the trunk muscles; both generate an increase in tissue loading. Taking all these findings into account will help clinician to provide adapted treatment for managing both pain and postural control.

## 1. Introduction

Chronic pain is defined by the International Association for Study of Pain (IASP) as “an unpleasant sensory and emotional experience associated with, or resembling that associated with, actual or potential tissue damage” [1] lasting more than 3 months [2]. By affecting more than 30% of the population worldwide [3], chronic pain is an economic burden and has a dramatic impact on biological, psychological, and sociological factors, resulting in poor quality of life [4,5,6]. Although medical care focuses on pain perception, psychological and functional disability should be considered [6]. It has been clearly demonstrated that pain interferes with sensorimotor control [7,8,9,10,11], and, more especially, with postural control [12,13,14,15,16,17,18,19,20].

Postural control, either in static or dynamic conditions, is an essential requirement to perform daily activities [21]. The upright standing human body, classically represented by an inverted pendulum model, is intrinsically unstable, as reflected by the movement of the center of mass (CoM) [22]. To maintain upright standing, the postural system requires efficient functioning of the sensorimotor mechanisms and the ability to detect body sways through reliable sensory systems integrating these sensory cues provided by the visual, vestibular, proprioceptive, and exteroceptive systems [23,24,25,26,27]. The integration of sensory information appears to be dynamically regulated based on available sensory information depending on environmental conditions, a process referred to as sensory reweighting [28,29,30,31]. When one (or more) of the sensory systems is altered, the Central Nervous System regulates balance by attributing a higher weight to the remaining afferent information [28]. Both sensory integration and reweighting are used by the neural control system to generate a corrective torque at the ankle to resist the deviations of the human body from an upright reference position [32]. Balance control, commonly assessed by measuring center of pressure (COP) displacement, could represent one of the sensorimotor control signatures observed in patients with chronic pain.

The main aim of this comprehensive review is to provide a synthesis of the effect of experimental and chronic pain on postural control by combining knowledge from the literature and identifying the potential impact of pain on the sensorimotor mechanisms involved in postural control. This review also summarizes the evidence supporting the importance of including postural control in the clinical assessment of patients suffering from chronic pain. Improving the knowledge of pain interference on postural control could help with developing new and adapted therapeutic approaches for patients presenting chronic pain.

## 2. From Nociception to Pain

The process leading to pain starts with the stimulation of nociceptors [33]. There are two main classes of nociceptors. The first comprises myelinated afferents of medium diameter (Aδ) that mediate acute and well-localized “first” or rapid pain. The second class of nociceptor consists of small diameter unmyelinated “C” fibers that transmit poorly localized “secondary” or slow pain [34]. Myelinated Aδ nociceptors respond to mechanical and thermal stimuli, whereas unmyelinated C-fiber polymodal nociceptors generally respond to mechanical, thermal, or chemical stimulation. Specific nociceptors are only excited when stimulus intensities reach the noxious range, suggesting that they possess biophysical and molecular properties allowing them to selectively detect and respond to harmful stimuli [34]. Ion channels on peripheral nociceptors can be activated by direct stimulation or by molecules released at a site of inflammation (bradykinin, prostaglandins, histamine, serotonin, and others), leading to the depolarization of small primary afferents of the first-order neurons expressing these channels [35]. Action potentials, with a frequency that is proportional to the intensity of the stimulus, propagate along the axons of myelinated or unmyelinated nociceptive fibers through the dorsal root ganglion (DRG) to the axonal endings of the spinal cord, which are organized into anatomically and electrophysiologically distinct laminae [33].

The nociceptive Aδ and C fibers surround the outermost layer of the dorsal horn. They enter the dorsal horn and end in the superficial layers (called Rexed Laminae I and II) or extend into the deep layers (Lamina V), probably via interneurons [34]. The lamina II plays a key role in the modulation of pain in the spinal cord [36,37]. The lamina II, also known as the substantia gelatinosa system, acts as an inhibitory mechanism on central transmission cell (T)-cells. The stimulation of nociceptive Aδ and C fibers inhibits the substantia gelatinosa cells, reducing the output and their inhibitory action on the (T)-cells, leading to an increase in their activity. The reduction in the ability of (T)-cells to receive or respond to the stimuli, is the hallmark of the gate control theory at the spinal level [38]. As a reminder, the (T)-cells are located in the dorsal horn of the spinal cord. They receive a balanced input of large of Aβ and small Aδ and C fibers activity in the peripheral nerves. Inhibitory interneurons, located in the substantia gelatinosa, can be activated by large afferents and can modulate the transmission of pain by projection to small fibers and central transmission cells [38].

Because pain is a complex multifactorial subjective experience, a large brain network is engaged during nociceptive processing. Numerous central nervous system structures (e.g., the anterior cingulate cortex, thalamus, and insula) consistently respond to transient nociceptive stimuli causing pain. The activation of this pain matrix or pain signature has been related to perceived pain intensity, both within and between individuals [39]. Following integration into the dorsal horn, nociceptive information is conducted via two phylogenetically distinct systems, the medial and the lateral systems, to the higher centers of the brainstem and brain. The medial system is involved in the affective and cognitive dimension of pain, pain memory, and autonomic responses [40,41,42]. This medial pathway projects directly to the higher brain structures and mainly includes the spinoreticular tract, the spinomesencephalic tract, the spinoparabrachial tracts, the spinohypothalamic tract, and the spinothalamic tract fibers. A component of the spinoreticular tract projects to the lateral reticular formation involved in motor control. The other component projects to the medial, pontomedullary reticular formation and, from there, to the thalamocortical circuits. A major target of the spinomesencephalic tract is the parabrachial nucleus of the pons, a region involving in the integration of the cardiovascular, autonomic, and motivational response to pain. Other collaterals of the spinohypothalamic pathway project at the thalamus and also innervate the medulla and pons of the brainstem, sites of origin of the descending modulatory pathways; please see [37]. The lateral system provides information on the location and duration of pain and plays an important role in the sensory-discriminating component of pain. This lateral system is formed by the spinocervical pathway, which projects to the lateral cervical nucleus at the C1–C3 level, and the nuclei of the dorsal column, which project to the cuneate and gracile nuclei of the dorsal column of the spinal cord. From the lateral cervical nucleus, information travels by the cervicothalamic tract to several thalamic nuclei, including the ventroposterior and posterior nucleus groups, and by a cervicomesencephalic pathway to the midbrain, including the periaqueducal grey and superior colliculus. With regard to the nuclei of the dorsal column, the output neurons project by the medial lemniscus to the ventroposterior and posterior groups of thalamic nuclei and to the superior colliculus; please see Millan [37].

## 3. Interaction between Pain and Postural Control

Experimental pain has been used to determine the potential impact of pain on balance control. By inducing heat pain on the lower leg muscle (45 °C), Blouin et al. [15] showed a significant increase in COP velocity in comparison with the non-pain condition (i.e., heat stimulation at 40 °C). Similarly, other studies have reported that a unilateral hypertonic saline injection at the infrapatellar fat pad [18], thigh [17], or leg muscles [16] led to significant increase in body sways and muscle activities. In addition, it has been reported that pain induced by electrical stimulation on the dorsum of the feet caused larger COP displacement [14]. By inducing different levels of pain (weak, moderate, extreme), the authors observed that the COP displacement scaled with the level of pain. Furthermore, they reported that pain induced on the hand did not change COP displacement, showing the specificity of the pain location related to postural control interference. The authors concluded the painful stimulation affects postural control via the sensorimotor mechanisms rather than cognitive processes related to perception of pain.

A systematic review, including 16 studies, reported that lower back pain (LBP) results in COP parameters alteration (i.e., increase in COP velocity and sway in anteroposterior direction) [19,20]. Pain influences the sensorimotor response in individuals with LBP, delaying and reducing the COP displacement on unstable surfaces [37], as well as increasing postural sway in the antero-posterior and medio-lateral direction in open eyes [21,43] and closed eyes [44] conditions, and in a single leg support [45]. Considering all of these result, pain may alter the sensorimotor components of the postural system controlling balance [46,47,48,49,50]. Pain and impaired postural control often imply reduced muscle strength [51], physical inactivity [52], and depression [53]. Musculoskeletal pain is also associated with an increased risk of falling [12,13,54]. Results from various studies also highlighted reduced trunk movements [55] and trunk stiffness [56]. These alterations likely cause postural instability [46] and may be an indicator of dysfunctional postural control strategies [56,57].

Some studies also reported a decrease in proprioceptive acuity, that is, patients with back or neck pain have less accurate positions sense [58,59] suggesting impairment in body sway perception. More specifically, Popa et al. [60] suggested that the deterioration of proprioceptive information of the lower limbs and the trunk determines a reduced accuracy in the sensory integration process, and thus, a more imprecise internal estimate of the center of mass (CoM) position in individuals with chronic lower back pain (CLBP). Consequently, the motor controller needs to increase the safety margin of the CoP shifts with respect to the predicted oscillation of the CoM, reflected by a greater sway. Individuals with CLBP might set ankle stiffness at a higher level in order to compensate for sensory deterioration [61], as already demonstrated by reduced plantar sole sensitivity [24,25]. The reweighting of proprioceptive input by increasing the gain at the ankle joints (increasing loading of ankle extensors by leaning more forward) may enhance sensory discrimination and help maintain a critical level of sensory information to adequately cope with postural perturbations [60]. Overall, these sensorimotor changes may alter postural control [62]. Balance disorders may be associated with specific clinical findings, such as reduced muscle strength, impaired cognition, sensory or motor deficits, lower-extremity myofascial trigger points [63], or change in flexibility and coordination [64]. Patients with chronic pain syndrome, such as fibromyalgia, reported larger body sway than healthy controls [65], and balance impairment represents one of the top 10 most debilitating symptoms [66]. It was proposed that fibromyalgia likely affects dynamic balance control because of altered somatosensory inputs to the central nervous system, including the abnormal perception of pain with light somatosensory stimulation [63].

Persistent pain also alters cognitive processes. As cognition contributes to balance control [67,68,69], it is crucial to assess the relationship between pain intensity, cognition, and balance control. Individuals with severe pain showed less effective executive functioning [70]. Such cognitive deficits are associated with impaired physical functioning including gait speed, balance performance, sit-to-stand, and trunk rotation [71]. Because pain alters the sensorimotor mechanisms involve in balance control [72,73], clinical evaluation should assess balance control.

## 4. Mechanisms of Action of Pain and Potential Mechanisms Involved in Postural Control Alteration in Pain Condition

Pain is intimately linked to the activation of a complex cerebral network, as mentioned above, and involves cortical reorganization. Results from studies inducing pain confirmed a causal relationship between pain and cortical changes [10,74,75,76,77]. Experimental pain studies showed an increase in the primary motor cortex (M1) activity [78,79,80]. Using electroencephalography (EEG), Stancák et al. [81] reported that short-lasting painful heat stimuli on the hand decreased beta (β: 15–30 Hz) activity within the sensorimotor cortex. Given the inhibitory role that β oscillations have on the motor cortex [82], the decrease in primary motor cortex (M1) activity suggests that a brief nociceptive stimulus could alter (reduction of the inhibition) the motor region, possibly to facilitate withdrawal responses [83]. In a recent systematic review and meta-analysis, Rohel et al. [10] confirmed the inhibitory effect of pain on corticospinal excitability. More specifically, Billot et al. [9] reported that heat pain applied at the tibialis anterior muscle significantly reduced corticospinal excitability either during active muscle contraction or at rest. These results provide evidence that nociceptive sensory input can impact corticospinal excitability at the lower limb. Incoming research using the transcranial magnetic stimulation of the lumbar erector spinae muscles [84] will help to delineate corticospinal excitability modulation with pain.

Using neuroimaging and neurostimulation, numerous studies showed that patients with chronic pain, such as complex regional pain syndrome (CRPS) [85] or phantom limb pain [86], presented cortical reorganization at the M1 level, with a smaller corticomotor representation of the affected limb, compared to pain-free participants. A normalization of the cortical changes was observed in CRPS patients following the administration of treatment over 1 to 6 months that consisted of graded sensorimotor retuning [87], once pain subsided [87,88], underlying the fact that cortical reorganization may play a major role in the physiopathology of chronic pain [87,88]. These results support a causal relationship between pain and cortical changes. The cerebrum works as an integrated system of circuits, and certain brain areas, other than those classically involved in pain perception and modulation, can be affected by nociceptive stimulations [83].

At a lower anatomical level, spinal control may likewise be affected by pain conditions. To date, experimental pain studies failed to provide strong evidence of a potential inhibitory or facilitatory effect at the spinal level [89]. Investigating the influence of pain (hypertonic saline into biceps brachii) at cortical and spinal levels, Martin et al. [90] showed that the cervicomedullary motor evoked potentials increased at rest for both biceps and triceps brachii, and for the agonist muscle during a constant level of elbow flexion (biceps) and extension (triceps). On the other hand, Le Pera et al. [49] reported reduced H-reflex amplitude reduction in the recovery period after related pain induced by a hypertonic saline injection in the flexor carpi radialis. The authors interpreted this delayed H-reflex depression by the inhibition of the spinal motoneurones excitability that overlaps the cortical inhibition observed by motor-evoked potential amplitude (corticospinal excitability) decrease. In addition to spinal excitability, pain induces steady variations in spinal transmission that could alter motor strategies [47]. For instance, prolonged exposure to nociceptive stimulations from the skin or sore muscles induced large errors in a torque-matching task [91]. The authors reported that participants overestimated the torque level generated by a limb affected by pain. In addition, pain could induce a distortion of the body image, leading to a biased estimation of the body position in space [50].

The assessment of motor control in patients presenting with CLBP considers three main classes of motor tasks, evaluating the control of the trunk in a steady-state condition (posture and movement) or challenging by predictable or unpredictable perturbations [92]. Regarding the first condition, the literature has provided inconsistent lumbar extensor muscle activity through 30 studies by reporting higher, no difference, or lower muscle activity [7]. The results may differ depending on the anatomical specificity of the muscle. For instance, for deeper muscles, there was a systematic inhibition, whereas for superficial muscles, activity was preferentially augmented [7]. Likewise, by investigating the anticipatory activation of the trunk muscles that occurred after expected or unexpected perturbations in CLBP patients, studies reported the late activation of the transversus abdominis and multifidus muscles [93,94,95,96,97,98], no modification [99], or earlier activation [100,101]. In line with these results, the trunk movement alteration observed in CLBP patients may result from proprioception deficiency [102,103]. Far from placing all these results in opposition, van Dieen et al. [92] propose to dichotomize patients’ profils/phenotypes, where one phenotype includes patients with tight trunk control associated with the over-activation of the trunk muscle due to excitability increase and causing tissue loading increase; and the second phenotype includes patients with loose control associated with excessive spinal movements due to excitability decrease and tissue loading increase. Thereby, in a nutshell, patients suffering from CLBP present an abnormal loading of the tissues in the lower back originating from different mechanisms.

## 5. Conclusions

There is no doubt that pain modifies movement and motor control, illustrated by postural control alteration. This review showed that both experimental and chronic pain lead to postural control impairments. Although cortical modification has been largely investigated with pain localized at the upper limb, cortical and spinal modulation focusing on spine and lower limb muscles have yet to be determined. Finally, different phenotypes of motor control by tight or loose trunk control should be considered to provide adapted treatment for managing both pain and postural control in patients presenting with chronic lower back pain.

## References

[1] Raja S.N., Carr D.B., Cohen M., Finnerup N.B., Flor H., Gibson S., Keefe F.J., Mogil J.S., Ringkamp M., Sluka K.A. (2020). The revised international association for the study of pain definition of pain: Concepts, challenges, and compromises. Pain.

[2] Treede R.D., Rief W., Barke A., Aziz Q., Bennett M.I., Benoliel R., Cohen M., Evers S., Finnerup N.B., First M.B. (2019). Chronic pain as a symptom or a disease: The iASP classification of chronic pain for the international classification of diseases (ICD-11). Pain.

[3] Cohen S.P., Vase L., Hooten W.M. (2021). Chronic pain: An update on burden, best practices, and new advances. Lancet.

[4] Naiditch N., Billot M., Moens M., Goudman L., Cornet P., Le Breton D., Roulaud M., Ounajim A., Page P., Lorgeoux B. (2021). Persistent spinal pain syndrome type 2 (PSPS-T2), a social pain? Advocacy for a social gradient of health approach to chronic pain. J. Clin. Med..

[5] Naiditch N., Billot M., Goudman L., Cornet P., Roulaud M., Ounajim A., Page P., Lorgeoux B., Baron S., Nivole K. (2021). Professional status of persistent spinal pain syndrome patients after spinal surgery (PSPS-T2): What really matters? A prospective study introducing the concept of « Adapted professional activity » inferred from clinical, psychological and social influence. J. Clin. Med..

[6] Ounajim A., Billot M., Louis P.Y., Slaoui Y., Frasca D., Goudman L., Roulaud M., Naiditch N., Lorgeoux B., Baron S. (2021). Finite mixture models based on pain intensity, functional disability and psychological distress composite assessment allow identification of two distinct classes of persistent spinal pain syndrome after surgery patients related to their quality of life. J. Clin. Med..

[7] Van Dieën J.H., Selen L.P.J., Cholewicki J. (2003). Trunk muscle activation in low back pain patients, an analysis of the literature. J. Electromyogr. Kinesiol..

[8] Hodges P.W., Tucker K. (2011). Moving differently in pain: A new theory to explain the adaptation to pain. Pain.

[9] Billot M., Neige C., Gagné M., Mercier C., Bouyer L.J. (2018). Effect of cutaneous heat pain on corticospinal excitability of the tibialis anterior at rest and during submaximal contraction. Neural Plast..

[10] Rohel A., Bouffard J., Patricio P., Mavromatis N., Billot M., Roy J.S., Bouyer L., Mercier C., Masse-Alarie H. (2021). The effect of experimental pain on the excitability of the corticospinal tract in humans: A systematic review and meta-analysis. Eur. J. Pain.

[11] Bank P.J.M., Peper C.E., Marinus J., Beek P.J., van Hilten J.J. (2013). Motor consequences of experimentally induced limb pain: A systematic review. Eur. J. Pain.

[12] Leveille S.G., Bean J., Bandeen-Roche K., Jones R., Hochberg M., Guralnik J.M. (2002). Musculoskeletal pain and risk for falls in older disabled women living in the community. J. Am. Geriatr. Soc..

[13] Foley S.J., Lord S.R., Srikanth V., Cooley H., Jones G. (2006). Falls risk is associated with pain and dysfunction but not radiographic osteoarthritis in older adults: Tasmanian older adult cohort study. Osteoarthr. Catilage.

[14] Corbeil P., Blouin J.S., Teasdale N. (2004). Effects of intensity and locus of painful stimulation on postural stability. Pain.

[15] Blouin J.S., Corbeil P., Teasdale N. (2003). Postural stability is altered by the stimulation of pain but not warm receptors in humans. BMC Musculoskelet. Disord..

[16] Hirata R.P., Arendt-Nielsen L., Graven-Nielsen T. (2010). Experimental calf muscle pain attenuates the postural stability during quiet stance and perturbation. Clin. Biomech..

[17] Hirata R.P., Ervilha U.F., Arendt-Nielsen L., Graven-Nielsen T. (2011). Experimental muscle pain challenges the postural stability during quiet stance and unexpected posture perturbation. J. Pain.

[18] Hirata R.P., Arendt-Nielsen L., Shiozawa S., Graven-Nielsen T. (2012). Experimental knee pain impairs postural stability during quiet stance but not after perturbations. Eur. J. Appl. Physiol..

[19] Ruhe A., Fejer R., Walker B. (2011). Is there a relationship between pain intensity and postural sway in patients with non-specific low back pain?. BMS Musculoskelet. Disord..

[20] Ruhe A., Fejer R., Walker B. (2011). Center of pressure excursion as a measure of balance performance in patients with non-specific low back pain compared to healthy controls: A systematic review of literature. Eur. Spine J..

[21] della Volpe R., Popa T., Ginanneschi F., Spidalieri R., Mazzocchio R., Rossi A. (2006). Changes in coordination of postural control during dynamic stance in chronic low back pain patients. Gait Posture.

[22] Loram I.D., Lakie M. (2002). Direct measurement of human ankle stiffness during quiet standing: The intrinsic mechanical stiffness is insufficient for stability. J. Physiol..

[23] Ivanenko Y., Gurfinkel V.S. (2018). Human Postural Control. Front. Neurosci..

[24] Billot M., Handrigan G.A., Simoneau M., Corbeil P., Teasdale N. (2013). Short term alteration of balance control after a reduction of plantar mechanoreceptor sensation through cooling. Neurosci. Lett..

[25] Billot M., Handrigan G.A., Simoneau M., Teasdale N. (2015). Reduced plantar sole sensitivity induces balance control modifications to compensate ankle tendon vibration and vision deprivation. J. Electromyogr. Kinesiol..

[26] Viseux F.J.F. (2020). The sensory role of the sole of the foot: Review and update on clinical perspectives. Neurophysiol. Clin..

[27] Viseux F., Lemaire A., Barbier F., Charpentier P., Leteneur S., Villeneuve P. (2019). How can the stimulation of plantar cutaneous receptors improve postural control? Review and clinical commentary. Neurophysiol. Clin..

[28] Peterka R.J. (2002). Sensorimotor integration in human postural control. J. Neurophysiol..

[29] Kiemel T., Oie K.S., Jeka J.J. (2002). Multisensory fusion and the stochastic structure of postural sway. Biol. Cybern..

[30] Oie K.S., Kiemel T., Jeka J.J. (2001). Human multisensory fusion of vision and touch: Detecting non-linearity with small changes in the sensory environment. Neurosci. Lett..

[31] Hwang S., Agada P., Kiemel T., Jeka J.J. (2014). Dynamic reweighting of three modalities for sensor fusion. PLoS ONE.

[32] Maurer C., Peterka R.J. (2005). A new interpretation of sponatenous sway measures based on a simple model of human postural control. J. Neurophysiol..

[33] Basbaum A.I., Jessell T., Kandel E.R., Schwartz J., Jessell T. (2000). The perception of Pain. Principles of Neuroscience.

[34] Basbaum A.I., Bautista D.M., Scherrer G., Julius D. (2009). Cellular and molecular mechanisms of pain. Cell.

[35] Woller S.A., Eddinger K.A., Corr M., Yaksh T.L. (2017). An overview of pathways encoding nociception. Clin. Exp. Rheumatol..

[36] Almeida T.F., Roizenblatt S., Tufik S. (2004). Afferent pain pathways: A neuroanatomical review. Brain Res..

[37] Millan M.J. (1999). The induction of pain: An integrative review. Prog. Neurobiol..

[38] Melzack R., Wall P.D. (1965). Pain mechanisms: A new theory. Science.

[39] Wager T.D., Atlas L.Y., Lindquist M.A., Roy M., Woo C.W., Kross E. (2013). An fMRI-based neurologic signature of physical pain. N. Engl. J. Med..

[40] De Ridder D., Adhia D., Vanneste S. (2021). The anatomy of pain and suffering in the brain and its clinical implications. Neurosci. Biobehav. Rev..

[41] Mouraux A., Iannetti G.D. (2018). The search for pain biomarkers in the human brain. Brain.

[42] Henry S.M., Hitt J.R., Jones S.L., Bunn J.Y. (2006). Decreased limits of stability in response to postural perturbations in subjects with low back pain. Clin. Biomech. (Bristol Avon).

[43] Hamaoui A., Do M.C., Bouisset S. (2004). Postural sway increase in low back pain subjects is not related to reduced spine range of motion. Neurosci. Lett..

[44] Mientjes M.I., Frank J.S. (1999). Balance in chronic low back pain patients compared to healthy people under various conditions in upright standing. Clin. Biomech. (Bristol Avon).

[45] Luoto S., Aalto H., Taimela S., Hurri H., Pyykkö I., Alaranta H. (1998). One-footed and externally disturbed two-footed postural control in patients with chronic low back pain and healthy control subjects. A controlled study with follow-up. Spine.

[46] Matre D.A., Sinkjaer T., Svensson P., Arendt-Nielsen L. (1998). Experimental muscle pain increases the human stretch reflex. Pain.

[47] Rossi A., Decchi B., Ginanneschi F. (1999). Presynaptic excitability changes of group Ia fibres to muscle nociceptive stimulation in humans. Brain Res..

[48] Capra N.F., Ro J.Y. (2000). Experimental muscle pain produces central modulation of proprioceptive signals arising from jaw muscle spindles. Pain.

[49] Le Pera D., Graven-Nielsen T., Valeriani M., Oliviero A., di Lazzaro V., Tonali P.A., Arendt-Nielsen L. (2001). Inhibition of motor system excitability at cortical and spinal level by tonic muscle pain. Clin. Neurophysiol..

[50] Gandevia S.C., Phegan C.M. (1999). Perceptual distortions of the human body image produced by local anaesthesia, pain and cutaneous stimulation. J. Physiol..

[51] Jadelis K., Miller M.E., Ettinger W.H., Messier S.P. (2001). Strength, balance, and the modifying effects of obesity and knee pain: Results from the observational arthritis study in senior (oasis). J. Am. Geriatr. Soc..

[52] Lamb S.E., Guralnik J.M., Buchner D.M., Ferrucci L.M., Hochberg M.C., Simonsick E.M., Fried L.P. (2000). Factors that modify the association between knee pain and mobility limitation in older women: The Women’s Health and aging study. Ann. Rheum. Dis..

[53] Hirvensalo M., Sakari-Rantala R., Kallinen M., Leinonen R., Lintunen T., Rantanen T. (2007). Underlying factors in the association between depressed mood and mobility limitation in older people. Gerontology.

[54] Lihavainen K., Sipilä S., Rantanen T., Sihvonen S., Sulkava R., Hartikainen S. (2010). Contribution of musculoskeletal pain to postural balance in community-dwelling people aged 75 years and older. J. Gerontol. A Biol. Sci. Med. Sci..

[55] Brumagne S., Janssens L., Janssens E., Goddyn L. (2008). Altered postural control in anticipation of postural instability in persons with reccurent low back pain. Gait Posture.

[56] Brumagne S., Janssens L., Knapen S., Clayes K., Suuden-Johanson E. (2008). Persons with recurrent low back pain exhibit a rigid postural control strategy. Eur. Spine J..

[57] Hides J.A., Richardson C.A., Jull G.A. (1996). Multifidus muscle recovery is not automatic after resolution of acute, first-episode low back pain. Spine.

[58] Moseley G.L., Hodges P.W. (2005). Are the changes in postural control associated with low back pain caused by pain interference?. Clin. J. Pain.

[59] Pinsault N., Vuillerme N., Pavan P. (2008). Cervicocephalic relocation test to the neutral head position: Assessment in bilateral labyrinthine-defective and chronic, nontraumatic neck pain patients. Arch. Phys. Med. Rehabil..

[60] Popa T., Bonifazi M., della Volpe R., Rossi A., Mazzocchio R. (2007). Adaptive changes in postural strategy selection in chronic low back pain. Exp. Brain Res..

[61] Casadio M., Morasso P.G., Sanguineti V. (2005). Direct measurement of ankle stiffness during quiet standing: Implications for control modelling and clinical application. Gait Posture.

[62] Brumagne S., Cordo P., Lysens R., Verschueren S., Swinnen S. (2000). The role of paraspinal muscle spindles in lumbosacral position sense in individuals with and without low back pain. Spine.

[63] Jones K.D., Horak F.B., Winters K.S., Morea J.M., Bennett R.M. (2009). Fibromyalgia is associated with impaired balance and falls. J. Clin. Rheumatol..

[64] Muto L.H.A., Sauer J.F., Yuan S.L.K., Sousa A., Mango P.C., Marques A.P. (2015). Postural control and balance self-efficacy in women with fibromyalgia: Are there differences?. Eur. J. Phys. Rehabil. Med..

[65] Trevisan D.C., Driusso P., Avila M.A., Gramani-Say K., Moreire F.M.A., Parizotto N.A. (2017). Static postural sway of women with and without fibromyalgia syndrome: A cross-sectional study. Clin. Biomech. (Bristol Avon).

[66] Bennett R.M., Jones J., Turk D.C., Russell I.J., Matallana L. (2007). An internet survey of 2596 people with fibromyalgia. BMC Musculoskelet. Disord..

[67] Pajala S., Era P., Koskenvuo M., Kaprio J., Tolvanen A., Rantanen T. (2007). Genetic and environmental contribution to postural balance of older women in single and dual task situations. Neurobiol. Aging.

[68] Shumway-Cook A., Woollacott M. (2000). Attentional demands and postural control: The effect of sensory context. J. Gerontol. A Biol. Sci. Med. Sci..

[69] Teasdale N., Simoneau M. (2001). Attentional demands for postural control: The effects of aging and sensory reintegration. Gait Posture.

[70] Karp J.F., Reynolds C.F., Butters M.A., Dew M.A., Mazumdar S., Begley A.E., Lenze E., Weiner D.K. (2006). The relationship between pain and mental flexibility in older adult pain clinic patients. Pain Med..

[71] Weiner D.K., Rudy T.E., Morrow L., Slaboda J., Lieber S. (2006). The relationship between pain, neuropsychological performance, and physical function in community-dwelling older adults with chronic low back pain. Pain Med..

[72] Sullivan E.V., Rose J., Rohlfing T., Pfefferbaum A. (2009). Postural sway reduction in aging men and women: Relation to brain structure, cognitive status, and stabilizing factors. Neurobiol. Aging.

[73] Treede R.D., Apkarian V.A., Bromm B., Greenspan J.D., Lenz F.A. (2000). Cortical representation of pain: Functional characterization of nociceptive areas near the lateral sulcus. Pain.

[74] Schabrun S.M., Jones E., Kloster J., Hodges P.W. (2013). Temporal association between changes in primary sensory cortex and corticomotor output during muscle pain. Neuroscience.

[75] Pelletier R., Higgins J., Bourbonnais D. (2017). The relationship of corticospinal excitability with pain, motor performance and disability in subjects with chronic wrist/hand pain. J. Electromyogr. Kinesiol..

[76] Goossens N., Rummens S., Janssens L., Caeyenberghs K., Brumagne S. (2018). Association between sensorimotor impairments and functional brain changes in patients with low back pain: A critical review. Am. J. Phys. Med. Rehabil..

[77] Goossens N., Janssens L., Brumagne S. (2019). Changes in the organization of the secondary somatosensory cortex while processing lumbar proprioception and the relationship with sensorimotor control in low back pain. Clin. J. Pain.

[78] Apkarian A.V., Gelnar P.A., Krauss B.R., Szeverenyi N.M. (2000). Cortical responses to thermal pain depend on stimulus size: A functional MRI study. J. Neurophysiol..

[79] Tracey I., Becerra L., Chang I., Breiter H., Jenkins L., Borsook D., Gonzalez R.G. (2000). Noxious hot and cold stimulation produce common patterns of brain activation in humans: A functional magnetic resonance imaging study. Neurosci. Lett..

[80] Burns E., Chipchase L.S., Schabrun S.M. (2016). Primary sensory and motor cortex function in response to acute muscle pain: A systematic review and meta-analysis. Eur. J. Pain.

[81] Stancak A., Polacek H., Vrana J., Mlynar J. (2007). Cortical oscillatory changes during warming and heating in humans. Neuroscience.

[82] Pogosyan A., Gaynor L.D., Eusebio A., Brown P. (2009). Boosting cortical activity at Beta-band frequencies slows movement in humans. Curr. Biol..

[83] Martel M., Harvey M.P., Houde F., Balg F., Goffaux P., Leonard G. (2017). Unravelling the effect of experimental pain on the corticomotor system using transcranial magnetic stimulation and electroencephalography. Exp. Brain Res..

[84] Desmons M., Rohel A., Desgagnés A., Mercier C., Massé-Alarie H. (2021). Influence of different transcranial magnetic stimulation current directions on the corticomotor control of lumbar erector spinae muscles during a static task. J. Neurophysiol..

[85] Krause P., Förderreuther S., Straube A. (2006). TMS motor cortical brain mapping in patients with complex regional pain syndrome type I. Clin. Neurophysiol..

[86] Flor H. (2003). Cortical reorganisation and chronic pain: Implications for rehabilitation. J. Rehabil. Med..

[87] Pleger B., Tegenthoff M., Ragert P., Förster A.F., Dinse H.R., Schwenkreis P., Nicolas V., Maier C. (2005). Sensorimotor returning [corrected] in complex regional pain syndrome parallels pain reduction. Ann. Neurol..

[88] Maihöfner C., Handwerker H.O., Neundörfer B., Birklein F. (2004). Cortical reorganization during recovery from complex regional pain syndrome. Neurology.

[89] Farina S., Valeriani M., Rosso T., Aglioti S., Tamburin S., Fiaschi A., Tinazzi M. (2001). Transient inhibition of the human motor cortex by capsaicin-induced pain. A study with transcranial magnetic stimulation. Neurosci. Lett..

[90] Martin P.G., Weerakkody N., Gandevia S.C., Taylor J.L. (2008). Group III and IV afferents differentially affect the motor cortex and motoneurones in humans. J. Physiol..

[91] Weerakkody N.S., Percival P., Canny B.J., Morgan D.L., Proske U. (2003). Force matching at the elbow joint is disturbed by muscle soreness. Somatosens. Mot. Res..

[92] Van Dieën J.H., Reeves N.P., Kawchuk G., van Dillen L.R., Hodges P.W. (2019). Motor control changes in low back pain: Divergence in presentations and mechanisms. J. Orthop. Sports Phys. Ther..

[93] Hodges P.W., Richardson C.A. (1996). Inefficient muscular stabilization of the lumbar spine associated with low back pain. A motor control evaluation of transversus abdominis. Spine.

[94] Hodges P.W., Richardson C.A. (1998). Delayed postural contraction of transversus abdominis in low back pain associated with movement of the lower limb. J. Spinal Disord..

[95] Hodges P.W., Richardson C.A. (1999). Altered trunk muscle recruitment in people with low back pain with upper limb movement at different speeds. Arch. Phys. Med. Rehabil..

[96] MacDonald D., Moseley L.G., Hodges P.W. (2009). Why do some patients keep hurting their back? Evidence of ongoing back muscle dysfunction during remission from recurrent back pain. Pain.

[97] Massé-Alarie H., Flamand V.H., Moffet H., Schneider C. (2012). Corticomotor control of deep abdominal muscles in chronic low back pain and anticipatory postural adjustments. Exp. Brain Res..

[98] Prins M.R., Griffioen M., Veeger T.T.J., Kiers H., Meijer O.G., van der Wurff P., Bruijn S.M., van Dieën J.H. (2018). Evidence of splinting in low back pain? A systematic review of perturbation studies. Eur. Spine J..

[99] Massé-Alarie H., Beaulieu L.D., Preuss R., Schneider C. (2015). Task-specificity of bilateral anticipatory activation of the deep abdominal muscles in healthy and chronic low back pain populations. Gait Posture.

[100] Gubler D., Mannion A.F., Schenk P., Gorelick M., Helbing D., Gerber H., Toma V., Sprott H. (2010). Ultrasound tissue doppler imaging reveals no delay in abdominal muscle feed-forward activity during rapid arm movements in patients with chronic low back pain. Spine.

[101] Moseley G.L., Hodges P.W. (2006). Reduced variability of postural strategy prevents normalization of motor changes induced by back pain: A risk factor for chronic trouble?. Behav. Neurosci..

[102] Tong M.H., Mousavi S.J., Kiers H., Ferreira P., Refshauge K., van Dieën J. (2017). Is there a relationship between lumbar proprioception and low back pain? A systematic review with meta-analysis. Arch. Phys. Med. Rehabil..

[103] Willigenburg N.W., Hoozemans M.J., van Dieën J.H. (2013). Precision control of trunk movement in low back pain patients. Hum. Mov. Sci..

